# Agroecology Can Promote Climate Change Adaptation Outcomes Without Compromising Yield In Smallholder Systems

**DOI:** 10.1007/s00267-023-01816-x

**Published:** 2023-04-01

**Authors:** Kyle M. Dittmer, Sabrina Rose, Sieglinde S. Snapp, Yodit Kebede, Sarah Brickman, Sadie Shelton, Cecelia Egler, Milena Stier, Eva Wollenberg

**Affiliations:** 1 Alliance of Bioversity International and the International Center for Tropical Agriculture (CIAT), Calí, Colombia; 2 International Maize and Wheat Improvement Center (CIMMYT), Texcoco, Mexico; 3 Michigan State University (MSU), East Lansing, MI USA; 4 French National Research Institute for Sustainable Development (IRD), Marseille, France; 5 University of Vermont (UVM), Burlington, VT USA; 6 The Gund Institute for Environment, University of Vermont, Burlington, VT USA

**Keywords:** Agroecology, Climate change mitigation, Climate change adaptation, Yield, Smallholder

## Abstract

A critical question is whether agroecology can promote climate change mitigation and adaptation outcomes without compromising food security. We assessed the outcomes of smallholder agricultural systems and practices in low- and middle-income countries (LMICs) against 35 mitigation, adaptation, and yield indicators by reviewing 50 articles with 77 cases of agroecological treatments relative to a baseline of conventional practices. Crop yields were higher for 63% of cases reporting yields. Crop diversity, income diversity, net income, reduced income variability, nutrient regulation, and reduced pest infestation, indicators of adaptative capacity, were associated with 70% or more of cases. Limited information on climate change mitigation, such as greenhouse gas emissions and carbon sequestration impacts, was available. Overall, the evidence indicates that use of organic nutrient sources, diversifying systems with legumes and integrated pest management lead to climate change adaptation in multiple contexts. Landscape mosaics, biological control (e.g., enhancement of beneficial organisms) and field sanitation measures do not yet have sufficient evidence based on this review. Widespread adoption of agroecological practices and system transformations shows promise to contribute to climate change services and food security in LMICs. Gaps in adaptation and mitigation strategies and areas for policy and research interventions are finally discussed.

## Introduction

Food systems contribute one-third of global emissions (18 GtCO_2_e [Crippa et al. 2021]) and are vulnerable to climate risks, so will need to change if they are to contribute to climate targets, while meeting food security and nutritional needs equitably (Steiner et al. 2020). Given its transformative nature and potential benefits for climate adaptation and mitigation, agroecology has entered climate change discourse as a potential pathway toward a more resilient and sustainable food system (IPCC 2022). Yet evidence for agroecology’s impacts on climate change is limited (Sinclair et al. 2019; Debray et al. 2019; Leippert et al. 2020; Aguilera et al. 2020; Bezner Kerr et al. 2021) and gaps in information persist (The High Level Panel of Experts on Food Security and Nutrition 2019). Our purpose is to synthesize literature on climate change mitigation and adaptation outcomes of agroecology in low- and middle-income countries (LMICs). According to Gliessman (2018), agroecology is “the integration of research, education, action, and change that brings sustainability to all parts of the food system: ecological, economic, and social.” The definition of agroecology has evolved since the early 1980s and continues to be redefined and contested (Gliessman 2018; The High Level Panel of Experts on Food Security and Nutrition 2019), but most fundamentally, agroecology promotes diverse farming systems, nutrient recycling, soil health, and biological pest control (The High Level Panel of Experts on Food Security and Nutrition 2019) and encompasses social dimensions such as farmer co-creation and knowledge sharing (Barrios et al. 2020).

In this paper we assess agroecological interventions’ impact on climate change adaptation, mitigation and yield outcomes relative to a baseline of conventional practices for smallholder agricultural systems in LMICs. We build on previous work by Snapp et al. (2021) by analyzing the direction and magnitude of these outcomes. We also identify knowledge gaps to inform investment and implementation of agroecological approaches. We hypothesized that: (i) agroecological interventions produce positive, statistically robust (*p* < 0.1) impacts for climate change adaptation and mitigation; (ii) yield trade-offs from agroecological interventions are minimal; (iii) evaluating yield effects for single crops alone does not adequately capture productivity effects of agroecological systems; (iv) land equivalence ratios (LER) are higher for agroecology relative to conventional systems; (v) agroecological interventions show different impacts depending on baseline input use and intensity of production; and (vi) agroecological interventions more often result in positive impacts where local adaptation of practices (e.g., local or indigenous knowledge, extension and education, altering technology by context, and involvement of farmer organization) is present.

## Methods

### Literature Search

**Fig. 1:**
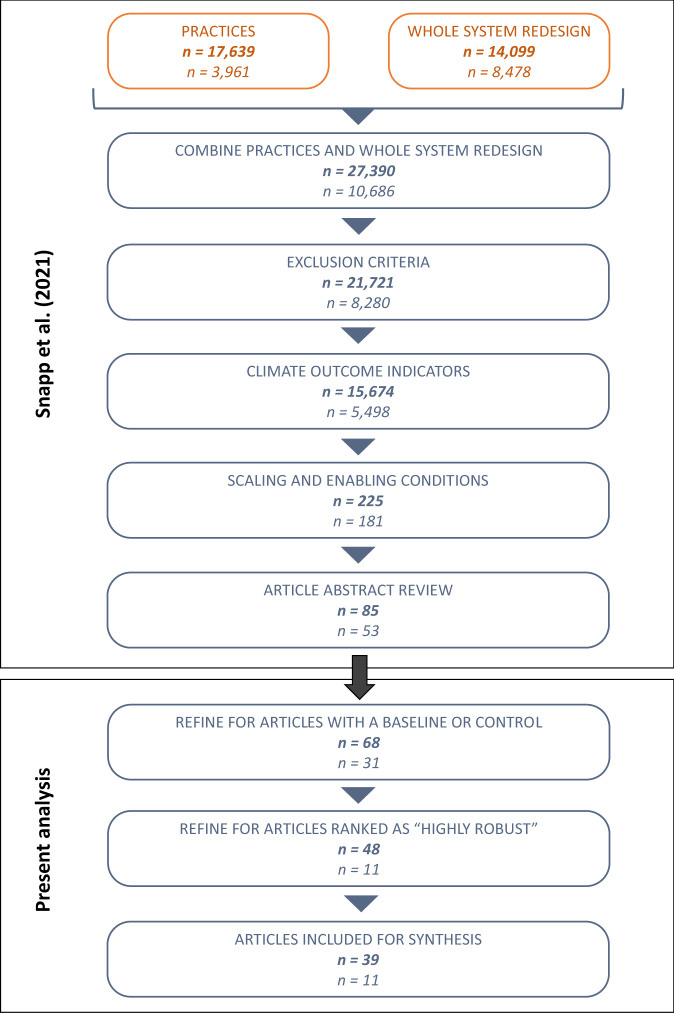
Stepwise procedure taken to identify and narrow candidate articles for nutrient management (bold text) and pest management in Snapp et al.^11^ and further refinement used for the present analysis

We reviewed published, peer-reviewed literature on nutrient management and pest and disease management in smallholder farms and collected data on 35 indicators of climate change mitigation, adaptation, and crop yield for agroecological interventions. Figure 1 shows the steps taken to identify candidate articles. We searched for articles that included key terms related to (i) agroecological practices that were used in LMICs, (ii) indicators of climate change mitigation, adaptation, or related co-benefits, and (iii) scaling up (Fig. 1). We excluded articles related only to agroecosystems or agro-ecological zones and articles that were meta-analyses, review, opinion, or perspective articles. The initial review resulted in 138 articles. See Snapp et al. (2021) for more information on literature search methodology and Supplementary Tables S1 and S2 for the Web of Science search strings used.

We reviewed the initial 138 articles to select articles with a clear baseline. Criteria for a clear baseline were: (i) a control, (ii) a counterfactual scenario, or (iii) a reference level for comparison. We then ranked studies’ robustness as high, medium or low based on (i) statistical analyses, (ii) reported p-values, (iii) experimental or quasi-experimental designs (no modeled data were included), (iv) spatial coverage, and (v) variability. Only articles that met at least one baseline criterion, were ranked as “high” for robustness (i.e., experimental designs or quasi-experimental designs with statistical analyses) and included at least one indicator of crop yield or climate change adaption or mitigation were included. The final set of articles represented studies mostly in Africa (70%), Asia (20%) and Latin America (6%) (Supplementary Table S3).

### Analysis

For each article, we recorded: (i) impacts of the agroecological cases on the 35 indicators for crop yield and climate change mitigation or adaptation (Supplementary Table S4); (ii) input levels for the baseline and agroecological case; and (iii) whether the case was developed using participatory or other processes involving local knowledge and learning. Aside from participatory processes, additional social dimensions of agroecology (e.g., equity or fairness, governance, land tenure) were out of scope for this analysis and were therefore not assessed. Data on additional social dimensions of agroecology were limited in the articles reviewed which focused more on biophysical impacts. An agroecological case was defined as a unique treatment (i.e., intervention) that evaluated the difference of an agroecological practice or system relative to a baseline at the start of the intervention. We recorded up to two cases per article to make the analysis manageable as some articles evaluated up to 25 cases. Where multiple cases existed, we selected the two cases that most strongly reflected agroecological practices based on Gliessman’s framework Gliessman (2016) and *The 10 Elements of Agroecology* developed by FAO (2018). Practices not considered agroecological according to these principles were not included. For example, if a study analyzed two nutrient amendment treatments such as the use of an organic nutrient source and an organic nutrient source plus synthetic fertilizer, only the former treatment was recorded. Overall, 77 unique agroecological cases were identified and evaluated.

Each agroecological case was assessed for its positive, negative, neutral, or mixed impact on the indicators. The response of a given indicator was recorded in a semi-quantitative way. Counts by category were carried out, where response was assigned to one of the following categories: The case had a positive (*p* < 0.1) impact on the indicator relative to the baseline; the case had a negative (*p* < 0.1) impact on the indicator relative to the baseline; the case did not have a statistically significant impact on the indicator (i.e., neutral); or the case had a mixed impact on the indicator (e.g., if an article analyzed both phosphorous and nitrogen uptake efficiency for the same case and one was positive and the other was negative). For crop yield, profitability, and greenhouse gas (GHG) emissions, we recorded the magnitude of change as well. For crop yield, we collected information on all crops that were relevant to the agroecological case, producing an additional 42 sub-cases (*n* = 119). We analyzed the frequency of indicator responses across articles.

We selected agroecological interventions as in Snapp et al. (2021). Interventions were selected as relevant to agroecological management of nutrients or pests and disease, or both. For nutrient management, interventions were searched for and considered as: agroforestry, organic farming, landscape mosaics (crop and off-crop habitats), livestock integration, organic nutrient source (manure, compost, green manure), legumes (intercrops, rotations, double legumes), crop diversity (variety studies or mixed cropping; no mention of legumes), conservation tillage, low input and mulch, regenerative agriculture (combination of any above) and ‘other’. For pest management, interventions were searched for and considered as: intercropping (non-push-pull), landscape structure (flower strips, trees integration), push-pull/companion crops, bioprotection (biopesticide, natural pesticide), biological control (enhancement of beneficial organisms), field sanitation measures, integrated pest management (IPM), organic farming, mechanical control, improved or reduced pesticide application, and ‘other’.

## Results

**Fig. 2:**
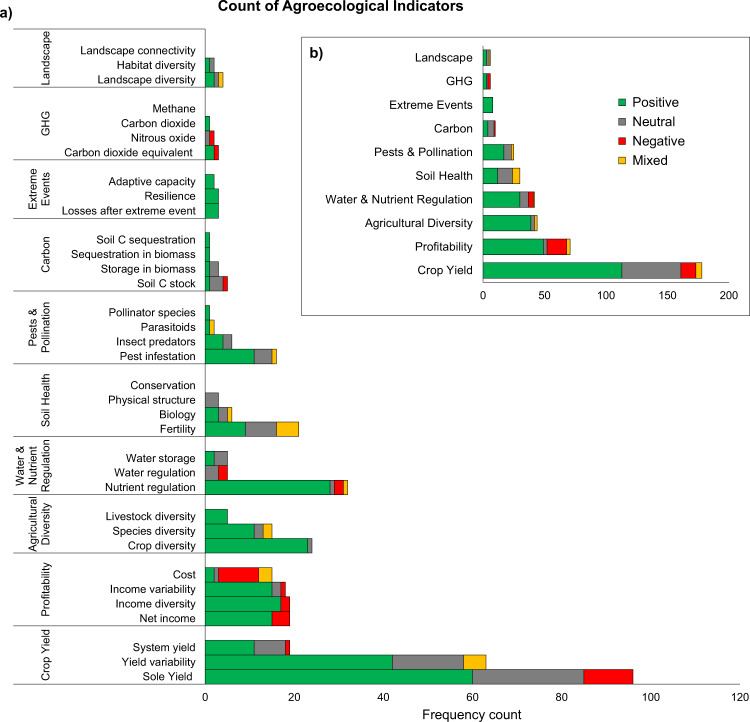
Frequency count reveals that agroecological interventions generally have a positive impact on productivity, profitability, and climate change adaptation co-benefits, though many outcomes are not well captured in the literature (i.e., landscapes, GHG emissions, extreme weather events and carbon stocks/sequestration). Number of reported responses (*n* = 178 for crop yield; *n* = 242 for climate change mitigation and adaptation) with a significant (*p* < 0.1) positive (green), significant negative (red), neutral (i.e., not significant finding; gray) and mixed (yellow) response to separate agroecological interventions indicators (**a**) and overall categories (**b**). Mitigation categories include GHG emissions (GHG) and carbon sequestration/storage (Carbon). Adaptation categories include diversification (Agricultural Diversity), response to extreme events (Extreme Events), landscape conservation (Landscape), pollination services and pest regulation (Pests & Pollination), Profitability, Soil Health, and Water & Nutrient Regulation

We analyzed 77 agroecological cases across the 50 articles for their impacts on 35 indicators (Supplementary Table S4), as a semi-quantitative systematic review building on Snapp et al. (2021). Of the cases evaluated in the review, the majority (*n* = 49, 64%) were assessed on one to four indicators relevant to our study. Where cases involved multiple crops, we also collected yield information for the crops under that case, which produced an additional 42 sub-cases (*n* = 119). This analysis generated 420 responses across all indicators, with 178 responses for crop yield indicators (42%) and 242 responses for both climate change mitigation and adaptation indicators (58%) (Fig. 2).

Of the 242 responses, adaptation accounted for 93% of responses (*n* = 226) whereas mitigation only accounted for 7% of responses (*n* = 16). Profitability was the most common indicator (29%), followed by agricultural diversity (18%) and water and nutrient regulation (17%) (Fig. 2b, Supplementary Table S5). Less common were soil health (12%), pests and pollination (10%), carbon sequestration/storage (4%), response to extreme weather events (3%), GHG emissions (2%) and landscapes and conservation (2%). The cases lacked responses for landscape connectivity, CH_4_ emissions, and soil conservation indicators (Fig. 2a).

### Climate Change Mitigation, Adaptation, and Yield Trends

We tested to see if positive, statistically robust (*p* < 0.1) impacts occur for both climate change mitigation and adaptation and crop yield due to agroecological interventions. Overall, our review shows that most climate change adaptation responses to agroecological cases reviewed were positive (*n* = 158 of 226, 70%) compared to their respective baselines (Fig. 2, Supplementary Table S5). The same was true for crop yield (*n* = 113 of 178, 63%). Climate change mitigation indicators had almost equal positive and neutral responses (44% positive, 38% neutral, 19% negative), however, evidence for mitigation comprised only 4% (*n* = 16) of all responses (Fig. 2b).

Agricultural diversity and water and nutrient regulation showed the most frequent evidence of positive responses to agroecological interventions (89% and 71% of cases, respectively) of the 10 categories evaluated in this review and presented in Fig. 2b. Crop yield, farm profitability, and pest and pollination categories also exhibited mostly positive responses (63%, 69% and 68%, respectively), while soil health showed equal shares of positive and neutral responses (40%). Response to extreme weather events, GHG emissions, landscapes, and carbon sequestration showed 100%, 50%, 50%, and 40% positive responses, respectively, though these categories were the least represented (Fig. 2b).

Of the 35 indicators, 23 showed mostly positive responses (Fig. 2a). The indicators with the strongest evidence for positive responses (~70% or higher) were crop diversity, income diversity, species diversity, net income, income variability, nutrient regulation, and pest infestation. Eight indicators included only positive responses but were among the least represented indicators (livestock diversity, pollinator species, sequestration in biomass, soil carbon sequestration, CO_2_ emissions, losses after extreme event, resilience, adaptive capacity). The cost indicator showed the strongest evidence for negative responses (60%), meaning cost often increased as a result of agroecological interventions. For cases that included an economic assessment (*n* = 20), we found that agroecological interventions increased costs by an average of $617 USD ha^−1^ relative to the baseline, however, net income increased by an average of $1050 USD ha^−1^ (Supplementary Table S7).

Our review highlights research gaps for indicators and practices. Generally, all climate change mitigation indicators were underrepresented and need further investigation. System yield and subsequent reporting of LER was another research gap, which limited our ability to test whether yield effects for single crops alone adequately capture productivity effects and whether LER are higher for agroecology relative to conventional systems. Of the 26 cases that evaluated system yield, only seven reported LER and four cases provided sufficient information to calculate LER (i.e., main and secondary crop yields reported). Such cases involved crop diversification, legumes (e.g., intercropping, rotation), agroforestry, and organic farming. Overall, LER ranged from 0.6 to 8.0, with the average being 1.8, indicating that one ha of a diversified system would produce the same yield as almost 2 ha of monoculture. The case for the lower limit represented two intercropping patterns, both consisting of maize and cowpea, but with a substitutive pattern developed by researchers compared to an additive pattern proposed by local farmers. The intervention for the upper limit represented monoculture maize without nutrient inputs compared to a cowpea maize double cropping system with an organic nutrient amendment.

### Agroecological System Design

**Fig. 3:**
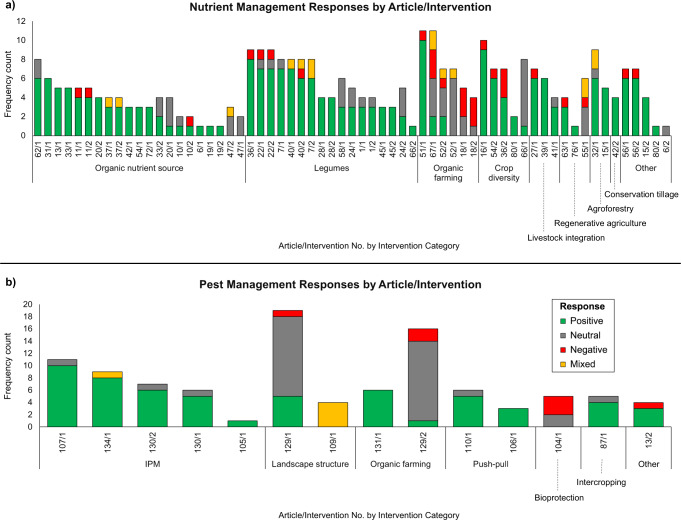
Frequency count of reported responses with a significant (*p* < 0.1) positive (green), significant negative (red), neutral (i.e., not significant finding; gray) and mixed (yellow) response to agroecological nutrient management intervention categories (**a**) and pest and disease management intervention categories (**b**). IPM = integrated pest management

To test whether agroecological system design improved climate impacts and increased yields, we examined interventions that reflected multiple practices or system-level shifts. Among nutrient management interventions, organic nutrient sources and legumes were the most prevalent (Fig. 3a), comprising 33% and 27% (*n* = 21 and 17, respectively). These intervention categories also showed the highest percentage of positive responses (79% and 75%, respectively). Conservation tillage, livestock integration and agroforestry also demonstrated a high proportion of positive responses (>80%), but were few in number (*n* = 1, 3 and 2, respectively). Organic farming had the highest variability in responses and highest proportion of negative responses (24% negative). Nutrient management interventions categorized as ‘other’ included strategic irrigation of rainfed crops and locally recommended synthetic fertilizer (N and P) rates. Landscape mosaics was the only intervention category not reported.

Pest and disease management interventions were less represented in our literature search, accounting for only 18% (*n* = 14) of the cases reviewed (Supplementary Table S6). IPM had the largest cumulative response count as well as the second greatest proportion of positive responses (88%) after push-pull/companion crop interventions (89% positive responses) (Fig. 3b). Biological control (e.g., enhancement of beneficial organisms), field sanitation measures, improved or reduced pesticide application and mechanical control were not represented within the screened literature. Bioprotection (e.g., biopesticide, natural pesticide, botanical pesticide) was only present in one of the 77 cases, yet demonstrated the greatest proportion of negative responses (60%). Like nutrient management interventions, organic farming was amongst the most variable intervention categories for pest management with 32% positive, 9% negative and 59% neutral responses. Landscape structure (e.g., flower strips, trees integration) also demonstrated variable results with responses being 22% positive, 4% negative, 57% neutral, and 17% mixed. Only one pest and disease management intervention was characterized as ‘other’.

### Intensification Level of Input Use

**Fig. 4:**
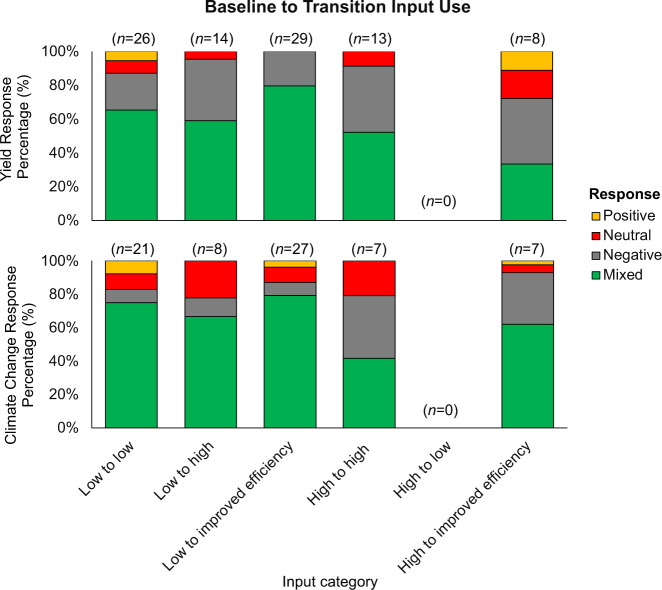
Percentage of responses across sole crop yield and climate change mitigation and adaptation indicators, normalized by the number of cases per input category, with a significant (*p* < 0.1) positive (green), significant negative (red), neutral (i.e., not significant finding; gray), and mixed (yellow) response for baseline inputs to agroecological cases inputs

We tested to see if agroecological cases’ impact on climate benefits varied with input use and intensity of production. We looked at crop yield and climate change mitigation and adaptation separately. For crop yield, the majority (77%) of case baselines had low inputs (Fig. 4), or no inputs. The cases with low-input baselines that transitioned to high input (intensification) with improved efficiency (e.g., locally recommended fertilizer rates) demonstrated the highest proportion of yield increases and positive yield responses (80%). This was the only input category without negative responses (Fig. 4). A small proportion (23%) of the case baselines had high inputs or did not specify the baseline input use (6%). High-input baselines with transitions to high input with improved efficiency cases demonstrated the highest proportion of yield decreases and negative yield responses (17%) and neutral responses (39%). High-input baselines with transitions to low-input were not reported for any cases.

Similar trends were observed for climate change mitigation and adaptation responses: a majority (73%) of cases had low-input baselines. Low-input baselines with transitions to high input with improved efficiency cases demonstrated the highest proportion of positive responses (79%). However, unlike yield responses, the low-input baseline to high-input (intensification) transitions demonstrated the highest proportion of negative responses (22%). Overall, cases with intensification generally led to more negative responses (~20% or higher) for climate change mitigation and adaptation relative to low or improved efficiency input transitions (Fig. 4). Potential trade-offs regarding the level of input use on crop yields and climate change outcomes must be considered in each specific context.

### Local Adaptation

We compared outcomes from agroecological cases with local adaptation (i.e., local or indigenous knowledge, extension and education, altering technology by context, and involvement of a farmer organization) to agroecological cases without local adaptation (i.e., present or absent). As is relates to social dimensions of agroecology, local adaptation through farmer participation, indigenous knowledge and co-development of technical options suited to local conditions may support more successful implementation and scaling up of practices with climate change adaptation and mitigation impacts while co-creation can enhance farmers’ adaptative capacity. Local adaptation was observed in 42 of the 77 cases and just under half of the articles and responses (23 of 50 articles, *n* = 200 of 420). Where local adaptation was present in agroecological cases, 56% of the 200 responses were positive, 30% were neutral, 9% were negative, and 6% were mixed. Where local adaptation was not present, 75% of the 220 responses were positive, 12% were neutral, 9% were negative, and 4% were mixed. We also observed a substantial number of responses of high costs where local adaptation was not present (Supplementary Fig. S1).

Several indicators were not equally represented across cases. Nutrient regulation and soil fertility impacts were reported more than twice as often in cases without local adaptation compared to those with local adaptation. Greenhouse gas emissions, soil physical structure, carbon storage and sequestration in biomass, soil carbon sequestration, water storage, water regulation, adaptive capacity and losses after extreme events were only observed in cases with local adaptation. Pollinator species and habitat diversity indicators were only observed in cases without local adaptation (Supplementary Fig. S1).

### Co-benefits

We tested for climate change co-benefits to yields by examining the association of climate change mitigation and adaptation with sole crop yield. The evidence was inconclusive based on our sample size. We found 145 responses for the subset of data that contained positive responses for sole yield, though the most frequent responses, and therefore the most evidence occurred for profitability (*n* = 44), water and nutrient regulation (*n* = 32) and agricultural diversity (*n* = 23) categories. The subset of data that contained negative sole crop yield with associated co-benefits captured a substantially smaller response count (*n* = 25). The impacts of agroecological interventions on climate change mitigation and adaptation co-benefits to yields is therefore a major research gap (Tamburini et al. 2020) in the studies reviewed.

## Discussion

This is the first systematic review we are aware of to assess the response of agroecological transitions on crop yield and climate change mitigation and adaptation outcomes in LMIC smallholder agricultural systems. We reviewed 50 articles, involving 77 unique agroecological cases. We found that agroecological cases were frequently associated with gains in crop yield, agricultural diversity, profitability, and water and nutrient regulation. The greatest body of evidence was associated with yield of a primary crop. Secondary crop yields were often not reported or were aggregated. This focus on primary crop yield is consistent with a review of sustainable intensification studies on smallholder farms (Reich et al. 2021). There was an evidence gap that prevented analysis of a broader range of trade-offs beyond these four indicators, with few systematic assessments of multiple dimensions, or for agroecology at large scales.

The trends observed in this review support the hypothesis that agroecological cases increase primary crop yield. This finding is consistent with a review that found most studies (78%, *n* = 56) had a positive relationship between a range of agroecological practices and food security and nutrition, where studies with more complex agroecological approaches (i.e., three or four agroecological components) were associated with a higher proportion of positive food security and nutrient outcomes (Bezner Kerr et al. 2021). Furthermore, a growing body of evidence (IPCC 2022) exists in favor of climate change adaptation and multiple co-benefits that are associated with practices and systems aligned with principles of agroecology, but benefits and trade-offs may vary by social and environmental contexts.

A high degree of context dependency of outcomes was observed. This is shown in the variability of outcomes associated with sole crop yields to overall whole system change (Fig. 2a) as well as the range of outcomes for low- versus high-input systems (Fig. 4), and is fully consistent with the literature (Tamburini et al. 2020; Reich et al. 2021). Our review shows that yield often decreases or stays the same with organic farming, likely as a result of being primarily low-input systems, but generally increases with legumes and organic nutrient sources (Supplementary Table S6). Yield stability improves in organic systems compared to conventional systems under enhanced fertility management (Knapp and van der Heijden 2018). Agroecological practices thus hold varying potential to increase or stabilize yields compared with conventional farming, depending on the context. Developing locally adapted cropping systems should take this context into account to identify agroecological solutions that enhance both crop yield and multiple ecosystem functions. Win-win outcomes may not always be feasible, as shown by a study on scaling organic field crop management which found that yields were reduced relative to conventional high-input management, particularly as field size increased (Kravchenko et al. 2017). Where agroecology achieves climate change adaptation, but lowers yields, farmers may be willing to forego some reduction to gain economic stability. However, where trade-offs between yields and climate change mitigation and adaptation are more extreme, policy incentives may be needed. There are societal interests in the environmental and climate change adaptation and mitigation services associated with organic and biologically based nutrient management that could be supported through subsidies and government interventions to overcome any yield penalties.

Indicators were not equally represented among articles with local adaptation of practices to farmers’ contexts and articles without (Supplementary Fig. S1). Subsequent comparisons were likely imbalanced as interventions, farmer characteristics, and other key factors varied across articles with and without local adaptation, though more climate change adaptation and mitigation indicators were generally reported when local adaptation was present relative to the absence. The value of participatory knowledge co-creation and dissemination via farmer-to-farmer approaches and advisory services is inherent to facilitate development, improvement, and uptake of agroecological practices. When supporting agroecology and promoting climate change resilience and mitigation, local adaptation is expected to help establish and strengthen functional knowledge and innovation systems (Leippert et al. 2020). Participatory approaches, including the participation of farmers and local communities in the co-design of projects and development of locally adapted interventions, building on local knowledge and respecting local belief systems and values, are needed for scaling up agroecology (Snapp et al. 2021). Scaling agroecology will also require a community-led bottom-up approach, collective actions, farmer-to-farmer extension system. As the performance of many technology options varies by social, economic and ecological context, a key need is to address innovation and knowledge transfer as an option by context interaction, i.e., the suitability of an intervention depends on the locally relevant context (Sinclair and Coe 2019; Leippert et al. 2020). Innovations in local learning should be evaluated and successful approaches promoted, such as information and communication technology informed campaigns and digital approaches to promote action learning (Heong et al. 2014).

Our study had some limitations. First, while considerable attention to the potential for agroecology to tackle climate change exists in LMICs (Leippert et al. 2020), our search did not result in many articles that provided specific and robust evidence for the comparative performance of agroecology with respect to a baseline production system (i.e., conventional farming). Thus, evidence on agroecology and climate change adaptation and mitigation with a clear agroecological focus and a reference baseline remains scarce. Second, the objectives of the articles analyzed were not in many cases explicitly focused on agroecological outcomes and climate change impacts. Yet, the outcomes were highly relevant and helped elucidate how agroecological transitions affect multiple climate change mitigation and adaptation indicators. Third, we selected only up to two interventions from each article, yet articles often contained more, with a wide range of variations in some cases. Our approach was based on the practicalities of a detailed assessment, and it did allow for a sample of the diversity of the agroecology approaches to be reviewed. Lastly, we did not include the large body of literature on agroecology that has been published in Portuguese and Spanish (Tomich et al. 2011).

Little evidence exists for agroecological interventions on climate change mitigation (see also Saj et al. [2017]), or resilience to extreme weather events other than for hurricanes in Central America (Holt-Giménez 2002). Soil carbon stocks were the most frequently observed form of mitigation, but had the highest variability amongst mitigation indicators and very few observations. There is currently almost no evidence on agroecology practices and GHG emissions (N_2_O and CH_4_) in LMICs or their mitigation, especially for livestock. Similarly, there is limited evidence on how to buffer effects of extreme weather events other than through agroforestry (Simelton et al. 2015; Sida et al. 2018) and crop diversification (Birthal and Hazrana 2019). The findings of this review indicate that although data on climate change mitigation and adaptation are not widely tracked for agroecological interventions, the studies reviewed here show that agroecological interventions promote significant positive climate change adaptation outcomes with no or minimal trade-offs for productivity and income.

The modest evidence for mitigation and resilience in this analysis highlights the need for further high-quality, long-term, research on farms and at landscape scales that compares agroecology against alternatives to better understand climate change mitigation co-benefits and resilience to extreme weather events and other climate risks. Such evidence can increase awareness of agroecology as a potential option for climate policies. Longer-term studies with innovative approaches such as on-farm benchmark studies, participatory modelling and community-engaged research are needed to understand climate change outcomes at multiple scales, while building capacity to adopt new practices. Policy research is also needed on how to achieve environmental services and other climate change outcomes at large scales without compromising productive services.

Based on these findings, evidence indicates that policy makers can prioritize the use of organic nutrient sources, diversifying systems with legumes and IPM for climate change adaptation outcomes to improve climate change adaptation in most contexts. Landscape mosaics, biological control (e.g., enhancement of beneficial organisms) and field sanitation measures do not yet have sufficient evidence based on this review. Linking funding and performance indicators in agriculture to environment and climate change outcomes can incentivize performance and be a practical way of addressing differences of opinion about what agroecology is. Nationally Determined Contributions (NDCs) may be one avenue. An analysis of the 2020–2022 NDCs showed, only 15 of 164 NDCs (9%) explicitly mentioned agroecology, with the emphasis clearly on adaptation. Only six countries included agroecology for climate change mitigation (Rose et al. 2021).

To address gaps identified in this synthesis, research priorities should include: (i) research in LMICs on climate change adaptation to extreme weather and quantitative assessment of GHG emissions and carbon sequestration/storage, (ii) scientific documentation of the effectiveness of agroecological approaches compared to alternatives, including performance in terms of environmental, social, and cost effectiveness, and (iii) evaluation of the impacts and lessons learned from programs currently implementing agroecology at scale to better understand local adaptation processes, across landscapes and regions, through agricultural development pathways that include agroecology.

## Supplementary information

Supplementary Information

## Data Availability

The data used in this analysis will be made publicly available via CGSpace (https://cgspace.cgiar.org).
