# Supplementary material for: Agroecology Can Promote Climate Change Adaptation Outcomes Without Compromising Yield In Smallholder Systems
Source: Environ Manage. 2023 Apr 1;72(2):333–42. doi: 10.1007/s00267-023-01816-x (PMC10287806; doi:10.1007/s00267-023-01816-x)
Supplement: Supplementary file 1 — Supplementary Information [file 267_2023_1816_MOESM1_ESM.docx]

**Table S1.** Search terms for nutrient management deep dive from Snapp et al. (2021). Note that TS refers to topic, TI refers to title.

| **Row** | **Search Terms** | **Description of Search Terms** |
| --- | --- | --- |
| 1 | TS=(nutrient* OR nitrogen OR phosphorus) | Only for Nutrient Management Deep Dive |
| 2 | TS=((intercrop* OR "crop association*" OR “doubled up” OR “doubled-up” OR “legume diver*” OR “rotat* diver*” OR “mixed crop*” OR "mixed cultivar*" OR "cover crop*" OR “green manure” OR “living cover”) AND (agricultur* OR farm* OR agroeco*)) | Cropping practices |
| 3 | TS= ((biofertili* OR "organic fertili*" OR manur* OR compost* OR mulch* OR "crop residue*" OR biopesticide* OR bioprotection OR "biological control" OR biocontrol*) AND (agricultur* OR farm* OR agroeco*)) | Soil amendments and pest management |
| 4 | TS= ((“perennial grain*” OR “push-pull” OR “recycl* nutrient*” OR “integrated crop” OR “integrated soil” OR “couple* carbon” OR “tighten nutrient” OR “nutrient budget”) AND (agricultur* OR farm* OR agroeco*)) | Pest management, nutrient recycling, and diversification |
| 5 | #1 AND (#2 OR #3 OR #4) | Combine search terms specific to the Nutrient Management Deep Dive with the search terms for Agroecological Practices |
| 6 | TS=((“crop livestock system” OR “integrated crop livestock” OR “crop-livestock” OR “agro-sylvo-pastoral” OR “sylvopastoral” OR “rotational grazing” OR diversif* OR “nutrient cycl*” OR “crop interaction” OR “pest-crop interaction” OR ecolog*) AND (agricultur* OR farm* OR agroeco*)) | Crop-livestock integration |
| 7 | TS=((“farmer participatory” OR “action research”) AND (agricultur* OR farm* OR agroeco*)) | Participatory action research |
| 8 | TS=((("*ecologic* intensification" OR “low input” OR permaculture OR holistic OR “integrated organic” OR “certified organic” OR agroforest*) AND (agricultur* OR farm* OR agroeco*) ) OR ((regenerative OR organic OR sustainab* OR agroeco*) NEAR/0 (agricultur* OR farm*))) | Organic/sustainable/regenerative agriculture, permaculture, or agroforestry |
| 9 | TS= (("integrated pest management" OR “landscape ecology” OR “landscape mosaic*” OR “landscape redesign”) AND (agricultur* OR farm* OR agroeco*)) | Landscape-level management |
| 10 | #1 AND (#6 OR #7 OR #8 OR #9) | Combine search terms specific to Nutrient Management Deep Dive with the search terms for Agroecological Systems |
| 11 | #5 OR #10 | Combine searches for Agroecological Practices and Agroecological Systems |
| 12 | TI=(USA OR US OR “United States” OR Canad* OR “North America*” OR Australia* OR “New Zealand” OR Europe* OR EU OR Austria* OR Belgium OR Belgian OR Bulgaria* OR Croatia* OR Cyprus OR Czech Republic OR Denmark OR Danish OR Estonia* OR Finland OR Finnish OR France OR French OR German* OR Greece OR Greek OR Hungar* OR Ireland OR Irish OR Ital* OR Latvia* OR Lithuania* OR Luxembourg OR Malta OR Netherlands OR Dutch OR Norw* OR Scandinavia* OR Poland OR Polish OR Portugal OR Portuguese OR Romania* OR Slovakia* OR Slovenia* OR Spain OR Spanish OR Swed* OR Switzerland OR Swiss OR “United Kingdom” OR UK OR Japan* OR Korea* OR Mediterranean) | High-income countries |
| 13 | TS=(agroecosystem* OR “agro-ecosystem*” OR “agroecological zone” OR “plastic mulch” OR “seed coating” OR soilless OR (wastewater NEAR/0 (municipal OR treatment))) | Agroecosystem, agroecological zones, and irrelevant agricultural practices |
| 14 | TI=(meta-analysis OR review OR opinion OR perspective) | Meta-analysis, reviews, and opinion articles |
| 15 | #11 NOT #12 NOT #13 NOT #14 | Exclude studies in high-income countries, articles on irrelevant topics, and meta-analysis and review articles |
| 16 | TS=("climate change mitigation” OR “greenhouse gas*” OR “carbon sequestration” OR “carbon storage” OR “sequester carbon” OR “store carbon” OR “carbon sink” OR “soil organic carbon” OR “carbon storage” OR “emissions* abatement” OR “emission* reduction” OR “reduced emission*” OR “low-emission* development” OR “nitrous oxide” OR “carbon dioxide” OR “methane") | Climate change mitigation |
| 17 | TS=("climate change adaptation”) | Climate change adaptation |
| 18 | TS=(productivity OR production OR yield OR co-product) | Productivity or yield |
| 19 | TS= (“crop diversity” OR “livestock diversity” OR “Ag* diversity” OR “genetic diversity” OR “Micro* diversity”) | Agricultural diversity |
| 20 | TS=(“Water regulation” OR “Water infiltration” OR “Nutrient regulation” OR “Soil water” OR “Soil nitrogen” OR “Soil aeration”) | Water or nutrient regulation |
| 21 | TS= ((Resilience OR Recovery) NEAR/4 (Hurricane OR Storm OR Extreme)) | Response to extreme event |
| 22 | TS=((health OR “organic matter” OR quality OR carbon OR aggregate* OR stability) NEAR/4 Soil) | Soil health |
| 23 | TS=((pollination OR pest OR arthropod* OR disease) NEAR (regulation OR service* OR management)) | Pollination services or pest regulation |
| 24 | TS=(carbon NEAR (biomass OR tree OR shrub OR grass OR grassland OR pasture OR rangeland OR agroforest* OR root*)) | Carbon sequestration in biomass |
| 25 | TS=((landscape* OR conservation) NEAR/4 (habitat OR diversity OR connectivity)) | Landscapes or conservation |
| 26 | TS=(“human capital” OR “traditional knowledge” OR “learning process*” OR “learning cycle*” OR “farm* learn*” OR “farmer exchange” OR “participatory extension” OR “citizen science” OR “living laborator*” OR “learning hub” OR “stakeholder engagement” OR “co-creation” OR “knowledge sharing”) | Adaptation via learning processes |
| 27 | #15 AND (#16 OR #17 OR #18 OR #19 OR #20 OR #21 OR #22 OR #23 OR #24 OR #25 OR #26) | Refine search results by requiring that they include the specified climate change indicators |
| 28 | TS=((smallhold* OR largehold*) OR ((small-scale OR large-scale OR medium-scale) NEAR/10 (farm* OR agricultur*))) | Smallholders and medium- and large-scale agriculture |
| 29 | #27 AND #28 | Refine search results by requiring that they reference farm size or agricultural scale |
| 30 | TS= (transition* OR scaling OR scale OR scale-up OR scale-out OR transform* OR adoption OR disadoption OR uptake OR innovation OR "institutional change" OR "organizational change" OR "systemic change" OR "social movement") | Scaling of agroecological practices or systems |
| 31 | TS= (drivers OR “enabling environment” OR “enabling conditions” OR barriers OR constraints OR obstacles) | Enabling conditions and barriers |
| 32 | TS= (intervention* OR development OR program* OR initiative* OR market* OR price* OR policy OR regulation OR governance OR subsidy OR "public-private" OR finance OR credit OR inputs OR "ecosystem payment*" OR "environmental payment*" OR "results-based payment*" OR "carbon market*" OR "ecosystem services market*" OR certification OR "land rights" OR tenure OR gender OR "food sovereignty" OR training OR "capacity building" OR "farmer field schools" OR education OR extension OR "co-learning" OR "innovation systems" OR participatory OR "action research" OR "seed systems" OR "consumer awareness" OR traceability OR collaboration OR "circular economy" OR stakeholder OR coordination OR organiz* OR "Value chain*") | Potential enabling conditions |
| 33 | #29 AND (#30 AND (#31 OR #32)) | Refine search results by requiring that they reference scaling or barriers to scaling |

**Table S2.** Search terms for pest and disease management deep dive from Snapp et al. (2021). Note that TS refers to topic, TI refers to title.

| **Row** | **Search Terms** | **Description of Search Terms** |
| --- | --- | --- |
| 1 | TS=(((Pest OR Disease OR Arthropod*) AND  (biopesticide* OR bioprotection OR "biological control" OR "natural pesticides" OR botanical* OR "beneficial arthropods" OR "Trap cropping" OR "Companion planting" OR “semiochemicals” OR biocontrol* OR “Push-pull” OR “Integrated crop”)) AND (agricultur* OR Farm* OR forest*)) | Only for Pest and Disease Management Deep Dive |
| 2 | TS=((intercrop* OR "crop association*" OR “doubled up” OR “doubled-up” OR “legume diver*” OR “rotat* diver*” OR “mixed crop*” OR "mixed cultivar*" OR "cover crop*" OR “green manure” OR “living cover”) AND (agricultur* OR farm* OR agroeco*)) | Cropping practices |
| 3 | TS= ((biofertili* OR "organic fertili*" OR manur* OR compost* OR mulch* OR "crop residue*" OR biopesticide* OR bioprotection OR "biological control" OR biocontrol*) AND (agricultur* OR farm* OR agroeco*)) | Soil amendments and pest management |
| 4 | TS= ((“perennial grain*” OR “push-pull” OR “recycl* nutrient*” OR “integrated crop” OR “integrated soil” OR “couple* carbon” OR “tighten nutrient” OR “nutrient budget”) AND (agricultur* OR farm* OR agroeco*)) | Pest management, nutrient recycling, and diversification |
| 5 | #1 AND (#2 OR #3 OR #4) | Combine search terms specific to the Pest Management Deep Dive with the search terms for Agroecological Practices |
| 6 | TS=((Pest OR Disease OR Arthropods) AND (agricultur* OR Farm* OR forest*)) | Only for Pest and Disease Management Deep Dive |
| 7 | TS=((“crop livestock system” OR “integrated crop livestock” OR “crop-livestock” OR “agro-sylvo-pastoral” OR “sylvopastoral” OR “rotational grazing” OR diversif* OR “nutrient cycl*” OR “crop interaction” OR “pest-crop interaction” OR ecolog*) AND (agriculture OR farm* OR agroeco*)) | Crop-livestock integration |
| 8 | TS=((“farmer participatory” OR “action research”) AND (agriculture OR farm* OR agroeco*)) | Participatory action research |
| 9 | TS=((("*ecologic* intensification" OR “low input” OR permaculture OR holistic OR “integrated organic” OR “certified organic” OR agroforest*) AND (agriculture OR farm* OR agroeco*) ) OR ((regenerative OR organic OR sustainab* OR agroeco*) NEAR/0 (agriculture OR farm*))) | Organic/sustainable/regenerative agriculture, permaculture, or agroforestry |
| 10 | TS= (("integrated pest management" OR “landscape ecology” OR “landscape mosaic*” OR “landscape redesign”) AND (agriculture OR farm* OR agroeco*)) | Landscape-level management |
| 11 | #6 AND (#7 OR #8 OR #9 OR #10) | Combine search terms specific to Pest Management Deep Dive with the search terms for Agroecological Systems |
| 12 | #5 OR #11 | Combine searches for Agroecological Practices and Agroecological Systems |
| 13 | TI=(USA OR US OR “United States” OR Canad* OR “North America*” OR Australia* OR “New Zealand” OR Europe* OR EU OR Austria* OR Belgium OR Belgian OR Bulgaria* OR Croatia* OR Cyprus OR Czech Republic OR Denmark OR Danish OR Estonia* OR Finland OR Finnish OR France OR French OR German* OR Greece OR Greek OR Hungar* OR Ireland OR Irish OR Ital* OR Latvia* OR Lithuania* OR Luxembourg OR Malta OR Netherlands OR Dutch OR Norw* OR Scandinavia* OR Poland OR Polish OR Portugal OR Portuguese OR Romania* OR Slovakia* OR Slovenia* OR Spain OR Spanish OR Swed* OR Switzerland OR Swiss OR “United Kingdom” OR UK OR Japan* OR Korea* OR Mediterranean) | High-income countries |
| 14 | TS=(agroecosystem* OR “agro-ecosystem*” OR “agroecological zone” OR “plastic mulch” OR “seed coating” OR soilless OR (wastewater NEAR/0 (municipal OR treatment))) | Agroecosystem, agroecological zones, and irrelevant agricultural practices |
| 15 | TI=(meta-analysis OR review OR opinion OR perspective) | Meta-analysis, reviews, and opinion articles |
| 16 | #12 NOT #13 NOT #14 NOT #15 | Exclude studies in high-income countries, articles on irrelevant topics, and meta-analysis and review articles |
| 17 | TS=("climate change mitigation” OR “greenhouse gas*” OR “carbon sequestration” OR “carbon storage” OR “sequester carbon” OR “store carbon” OR “carbon sink” OR “soil organic carbon” OR “carbon storage” OR “emissions* abatement” OR “emission* reduction” OR “reduced emission*” OR “low-emission* development” OR “nitrous oxide” OR “carbon dioxide” OR “methane") | Climate change mitigation |
| 18 | TS=("climate change adaptation”) | Climate change adaptation |
| 19 | TS=(productivity OR production OR yield OR co-product) | Productivity or yield |
| 20 | TS= (“crop diversity” OR “livestock diversity” OR “Ag* diversity” OR “genetic diversity” OR “Micro* diversity”) | Agricultural diversity |
| 21 | TS=(“Water regulation” OR “Water infiltration” OR “Nutrient regulation” OR “Soil water” OR “Soil nitrogen” OR “Soil aeration”) | Water or nutrient regulation |
| 22 | TS= ((Resilience OR Recovery) NEAR/4 (Hurricane OR Storm OR Extreme)) | Response to extreme event |
| 23 | TS=((health OR “organic matter” OR quality OR carbon OR aggregate* OR stability) NEAR/4 Soil) | Soil health |
| 24 | TS=((pollination OR pest OR arthropod* OR disease) NEAR (regulation OR service* OR management)) | Pollination services or pest regulation |
| 25 | TS=(carbon NEAR (biomass OR tree OR shrub OR grass OR grassland OR pasture OR rangeland OR agroforest* OR root*)) | Carbon sequestration in biomass |
| 26 | TS=((landscape* OR conservation) NEAR/4 (habitat OR diversity OR connectivity)) | Landscapes or conservation |
| 27 | TS=(“human capital” OR “traditional knowledge” OR “learning process*” OR “learning cycle*” OR “farm* learn*” OR “farmer exchange” OR “participatory extension” OR “citizen science” OR “living laborator*” OR “learning hub” OR “stakeholder engagement” OR “co-creation” OR “knowledge sharing”) | Adaptation via learning processes |
| 28 | #16 AND (#17 OR #18 OR #19 OR #20 OR #21 OR #22 OR #23 OR #24 OR #25 OR #26 OR #27) | Refine search results by requiring that they include the specified climate change indicators |
| 29 | TS=((smallhold* OR largehold*) OR ((small-scale OR large-scale OR medium-scale) NEAR/10 (farm* OR agricultur*))) | Smallholders and medium- and large-scale agriculture |
| 30 | #28 AND #29 | Refine search results by requiring that they reference farm size or agricultural scale |
| 31 | TS= (transition* OR scaling OR scale OR scale-up OR scale-out OR transform* OR adoption OR disadoption OR uptake OR innovation OR "institutional change" OR "organizational change" OR "systemic change" OR "social movement") | Scaling of agroecological practices or systems |
| 32 | TS= (drivers OR “enabling environment” OR “enabling conditions” OR barriers OR constraints OR obstacles) | Enabling conditions and barriers |
| 33 | TS= (intervention* OR development OR program* OR initiative* OR market* OR price* OR policy OR regulation OR governance OR subsidy OR "public-private" OR finance OR credit OR inputs OR "ecosystem payment*" OR "environmental payment*" OR "results-based payment*" OR "carbon market*" OR "ecosystem services market*" OR certification OR "land rights" OR tenure OR gender OR "food sovereignty" OR training OR "capacity building" OR "farmer field schools" OR education OR extension OR "co-learning" OR "innovation systems" OR participatory OR "action research" OR "seed systems" OR "consumer awareness" OR traceability OR collaboration OR "circular economy" OR stakeholder OR coordination OR organiz* OR "Value chain*") | Potential enabling conditions |
| 34 | #30 AND (#31 AND (#32 OR #33)) | Refine search results by requiring that they reference scaling or barriers to scaling |

| **Table S3:** Descriptive summary statistics for pest and nutrient management articles reviewed. | | |
| --- | --- | --- |
| **Feature** | | **Count** |
| Continent | Africa | 35 |
|  | Asia | 10 |
|  | Latin America | 3 |
|  | Multiple | 2 |
|  | *Total* | *50* |
| Study Methods | Experiment | 36 |
|  | Survey | 2 |
|  | Secondary data | 0 |
|  | Mixed methods | 12 |
|  | *Total* | *50* |
| Farm Size | Small | 40 |
|  | Medium | 1 |
|  | Large | 1 |
|  | Mix | 2 |
|  | Not specified | 6 |
|  | *Total* | *50* |
| Baseline  Input Use | Low | 56 |
|  | High | 14 |
|  | High, improved efficiency | 2 |
|  | Not specified | 5 |
|  | *Total* | *77* |
| Intervention  Input Use | Low | 21 |
|  | High | 15 |
|  | High, improved efficiency | 36 |
|  | Not specified | 5 |
|  | *Total* | *77* |
| Local Adaptation | Present | 35 |
|  | Absent | 42 |
|  | *Total* | *77* |

| **Table S4:** Crop yield, climate change mitigation and adaptation categories and associated indicators. | |
| --- | --- |
| **Category** | **Indicator** |
| Crop yield | Sole yield (individual crops) |
|  | System yield (crops grown in the same system) |
|  | Yield variability (trends amongst multiple site/years) |
| *Mitigation* | |
| GHG emissions | Carbon dioxide equivalent |
|  | Carbon dioxide |
|  | Methane |
|  | Nitrous oxide |
| Carbon (C) sequestration/storage | C storage in soil |
|  | C storage in biomass |
|  | C sequestration in soil |
|  | C sequestration in biomass |
| *Adaptation* | |
| Agricultural diversity | Crop diversity |
|  | Livestock diversity |
|  | Species diversity |
| Response to extreme events | Impact on losses after extreme event |
|  | Resilience |
|  | Adaptative capacity |
| Landscape and conservation | Habitat diversity |
|  | Landscape diversity |
|  | Landscape connectivity |
| Pollination services and pest regulation | Insect predators |
|  | Pollinator species |
|  | Parasitoids |
|  | Pest infestation |
| Profitability | Net income |
|  | Cost |
|  | Income diversity |
|  | Income variability |
| Soil health | Soil conservation |
|  | Soil physical structure |
|  | Soil fertility |
|  | Soil biology |
| Water and nutrient regulation | Water regulation |
|  | Water storage |
|  | Nutrient regulation |

| **Table S5:** Responses for climate change mitigation, adaptation, and crop yield indicators with associated significance. | | | | | | | |
| --- | --- | --- | --- | --- | --- | --- | --- |
| **Category** | **Indicator** | **Significance** | **Response Count** | | | | |
|  |  |  | *Positive* | *Negative* | *Neutral* | *Mixed* | *Total* |
| Agricultural diversity | Crop diversity | Significant | 19 | 0 | 0 | 0 | *19* |
|  |  | Not significant | 0 | 0 | 0 | 0 | *0* |
|  |  | Not specified | 4 | 0 | 1 | 0 | *5* |
|  | Livestock diversity | Significant | 4 | 0 | 0 | 0 | *4* |
|  |  | Not significant | 0 | 0 | 0 | 0 | *0* |
|  |  | Not specified | 1 | 0 | 0 | 0 | *1* |
|  | Species diversity | Significant | 10 | 0 | 0 | 0 | *10* |
|  |  | Not significant | 0 | 0 | 2 | 0 | *2* |
|  |  | Not specified | 1 | 0 | 0 | 2 | *3* |
| Carbon | Carbon storage in biomass | Significant | 1 | 0 | 0 | 0 | *1* |
|  |  | Not significant | 0 | 0 | 2 | 0 | *2* |
|  |  | Not specified | 0 | 0 | 0 | 0 | *0* |
|  | Soil carbon stock | Significant | 0 | 1 | 0 | 0 | *1* |
|  |  | Not significant | 0 | 0 | 3 | 0 | *3* |
|  |  | Not specified | 1 | 0 | 0 | 0 | *1* |
|  | Carbon sequestration in biomass | Significant | 0 | 0 | 0 | 0 | *0* |
|  |  | Not significant | 0 | 0 | 0 | 0 | *0* |
|  |  | Not specified | 1 | 0 | 0 | 0 | *1* |
|  | Soil carbon sequestration | Significant | 0 | 0 | 0 | 0 | *0* |
|  |  | Not significant | 0 | 0 | 0 | 0 | *0* |
|  |  | Not specified | 1 | 0 | 0 | 0 | *1* |
| Extreme events | Impact on losses | Significant | 0 | 0 | 0 | 0 | *0* |
|  |  | Not significant | 0 | 0 | 0 | 0 | *0* |
|  |  | Not specified | 3 | 0 | 0 | 0 | *3* |
|  | Resilience | Significant | 2 | 0 | 0 | 0 | *2* |
|  |  | Not significant | 0 | 0 | 0 | 0 | *0* |
|  |  | Not specified | 1 | 0 | 0 | 0 | *1* |
|  | Adaptative capacity | Significant | 0 | 0 | 0 | 0 | *0* |
|  |  | Not significant | 0 | 0 | 0 | 0 | *0* |
|  |  | Not specified | 2 | 0 | 0 | 0 | *2* |
| Greenhouse  gas emissions | CO_2_e | Significant | 0 | 1 | 0 | 0 | *1* |
|  |  | Not significant | 0 | 0 | 0 | 0 | *0* |
|  |  | Not specified | 2 | 0 | 0 | 0 | *2* |
|  | CO_2_ | Significant | 1 | 0 | 0 | 0 | *1* |
|  |  | Not significant | 0 | 0 | 0 | 0 | *0* |
|  |  | Not specified | 0 | 0 | 0 | 0 | *0* |
|  | CH_4_ | Significant | 0 | 0 | 0 | 0 | *0* |
|  |  | Not significant | 0 | 0 | 0 | 0 | *0* |
|  |  | Not specified | 0 | 0 | 0 | 0 | *0* |
|  | N_2_O | Significant | 0 | 1 | 0 | 0 | *1* |
|  |  | Not significant | 0 | 0 | 1 | 0 | *1* |
|  |  | Not specified | 0 | 0 | 0 | 0 | *0* |
| Landscape | Habitat diversity | Significant | 1 | 0 | 0 | 0 | *1* |
|  |  | Not significant | 0 | 0 | 1 | 0 | *1* |
|  |  | Not specified | 0 | 0 | 0 | 0 | *0* |
|  | Landscape diversity | Significant | 2 | 0 | 0 | 1 | *3* |
|  |  | Not significant | 0 | 0 | 1 | 0 | *1* |
|  |  | Not specified | 0 | 0 | 0 | 0 | *0* |
|  | Landscape connectivity | Significant | 0 | 0 | 0 | 0 | *0* |
|  |  | Not significant | 0 | 0 | 0 | 0 | *0* |
|  |  | Not specified | 0 | 0 | 0 | 0 | *0* |
| Pollination | Insect predators | Significant | 4 | 0 | 0 | 0 | *4* |
|  |  | Not significant | 0 | 0 | 2 | 0 | *2* |
|  |  | Not specified | 0 | 0 | 0 | 0 | *0* |
|  | Pollinator species | Significant | 0 | 0 | 0 | 0 | *0* |
|  |  | Not significant | 0 | 0 | 0 | 0 | *0* |
|  |  | Not specified | 1 | 0 | 0 | 0 | *1* |
|  | Parasitoids | Significant | 1 | 0 | 0 | 1 | *2* |
|  |  | Not significant | 0 | 0 | 0 | 0 | *0* |
|  |  | Not specified | 0 | 0 | 0 | 0 | *0* |
|  | Pest infestation | Significant | 10 | 0 | 0 | 1 | *11* |
|  |  | Not significant | 0 | 0 | 4 | 0 | *4* |
|  |  | Not specified | 1 | 0 | 0 | 0 | *1* |
| Crop yield | Sole yield | Significant | 46 | 9 | 0 | 0 | *55* |
|  |  | Not significant | 0 | 0 | 24 | 0 | *24* |
|  |  | Not specified | 14 | 2 | 1 | 0 | *17* |
|  | System yield | Significant | 6 | 1 | 0 | 0 | *7* |
|  |  | Not significant | 0 | 0 | 7 | 0 | *7* |
|  |  | Not specified | 5 | 0 | 0 | 0 | *5* |
|  | Yield variability | Significant | 32 | 0 | 0 | 2 | *34* |
|  |  | Not significant | 0 | 0 | 16 | 2 | *18* |
|  |  | Not specified | 10 | 0 | 0 | 1 | *11* |
| Profitability | Net income | Significant | 6 | 0 | 0 | 0 | *6* |
|  |  | Not significant | 0 | 0 | 0 | 0 | *0* |
|  |  | Not specified | 9 | 4 | 0 | 0 | *13* |
|  | Cost | Significant | 0 | 1 | 0 | 3 | *4* |
|  |  | Not significant | 0 | 0 | 1 | 0 | *1* |
|  |  | Not specified | 2 | 8 | 0 | 0 | *10* |
|  | Income diversity | Significant | 15 | 1 | 0 | 0 | *16* |
|  |  | Not significant | 0 | 0 | 0 | 0 | *0* |
|  |  | Not specified | 2 | 1 | 0 | 0 | *3* |
|  | Income variability | Significant | 3 | 0 | 0 | 0 | *3* |
|  |  | Not significant | 0 | 0 | 2 | 0 | *2* |
|  |  | Not specified | 12 | 1 | 0 | 0 | *13* |
| Soil health | Conservation | Significant | 0 | 0 | 0 | 0 | *0* |
|  |  | Not significant | 0 | 0 | 0 | 0 | *0* |
|  |  | Not specified | 0 | 0 | 0 | 0 | *0* |
|  | Physical structure | Significant | 0 | 0 | 0 | 0 | *0* |
|  |  | Not significant | 0 | 0 | 0 | 0 | *0* |
|  |  | Not specified | 0 | 0 | 3 | 0 | *3* |
|  | Fertility | Significant | 6 | 0 | 0 | 2 | *8* |
|  |  | Not significant | 0 | 0 | 0 | 1 | *1* |
|  |  | Not specified | 3 | 0 | 7 | 2 | *12* |
|  | Biology | Significant | 3 | 0 | 0 | 0 | *3* |
|  |  | Not significant | 0 | 0 | 1 | 0 | *1* |
|  |  | Not specified | 0 | 0 | 1 | 1 | *2* |
| Water and nutrient  regulation | Water regulation | Significant | 0 | 0 | 0 | 0 | *0* |
|  |  | Not significant | 0 | 0 | 3 | 0 | *3* |
|  |  | Not specified | 0 | 2 | 0 | 0 | *2* |
|  | Water storage | Significant | 0 | 0 | 0 | 0 | *0* |
|  |  | Not significant | 0 | 0 | 3 | 0 | *3* |
|  |  | Not specified | 2 | 0 | 0 | 0 | *2* |
|  | Nutrient regulation | Significant | 22 | 2 | 0 | 0 | *24* |
|  |  | Not significant | 0 | 0 | 1 | 0 | *1* |
|  |  | Not specified | 6 | 0 | 0 | 1 | *7* |

| **Table S6:** Nutrient management and pest and disease management **r**esponses by agroecological intervention type. Mitigation categories include GHG emissions and carbon sequestration/storage. Adaptation categories include diversification, response to extreme events, landscape conservation, pollination services and pest regulation, profitability, soil health and water and nutrient regulation. | | | | | | | | | | | | |
| --- | --- | --- | --- | --- | --- | --- | --- | --- | --- | --- | --- | --- |
| Nutrient Management | **Mitigation** | | | | **Adaptation** | | | | **Crop yield** | | | |
|  | *Positive* | *Neutral* | *Negative* | *Mixed* | *Positive* | *Neutral* | *Negative* | *Mixed* | *Positive* | *Neutral* | *Negative* | *Mixed* |
| Agroforestry | 0 | 0 | 0 | 0 | 7 | 0 | 0 | 0 | 4 | 1 | 0 | 2 |
| Organic farming | 0 | 0 | 0 | 0 | 12 | 6 | 3 | 2 | 2 | 10 | 8 | 2 |
| Livestock integration | 2 | 0 | 1 | 0 | 5 | 0 | 0 | 0 | 8 | 1 | 0 | 0 |
| Organic nutrient source | 3 | 2 | 1 | 0 | 21 | 2 | 2 | 3 | 33 | 8 | 0 | 0 |
| Legumes | 0 | 0 | 0 | 0 | 48 | 5 | 3 | 3 | 29 | 8 | 1 | 1 |
| Crop diversity | 0 | 0 | 0 | 0 | 11 | 0 | 3 | 0 | 11 | 7 | 2 | 0 |
| Conservation tillage | 0 | 0 | 0 | 0 | 2 | 0 | 0 | 0 | 2 | 0 | 0 | 0 |
| Regenerative agriculture | 0 | 0 | 1 | 0 | 3 | 3 | 1 | 2 | 1 | 0 | 0 | 0 |
| Other | 2 | 0 | 0 | 0 | 11 | 0 | 2 | 0 | 4 | 1 | 0 | 0 |
| Pest Management | **Mitigation** | | | | **Adaptation** | | | | **Crop yield** | | | |
|  | *Positive* | *Neutral* | *Negative* | *Mixed* | *Positive* | *Neutral* | *Negative* | *Mixed* | *Positive* | *Neutral* | *Negative* | *Mixed* |
| Intercropping | 0 | 0 | 0 | 0 | 4 | 1 | 0 | 0 | 0 | 0 | 0 | 0 |
| Landscape structure | 0 | 2 | 0 | 0 | 5 | 5 | 1 | 4 | 0 | 6 | 0 | 0 |
| Push-pull/companion cropping | 0 | 0 | 0 | 0 | 5 | 1 | 0 | 0 | 3 | 0 | 0 | 0 |
| Bioprotection | 0 | 0 | 0 | 0 | 0 | 1 | 3 | 0 | 0 | 1 | 0 | 0 |
| IPM | 0 | 0 | 0 | 0 | 14 | 3 | 0 | 1 | 16 | 0 | 0 | 0 |
| Organic farming | 0 | 2 | 0 | 0 | 7 | 6 | 2 | 0 | 0 | 5 | 0 | 0 |
| Other | 0 | 0 | 0 | 0 | 3 | 0 | 0 | 0 | 0 | 0 | 1 | 0 |

| **Table S7:** Profitability summary statistics by individual interventions and intervention type. For statistical significance, yes = statistically significant at p < 0.1, no = not statistically significant. NA = not applicable. | | | | | | | | | |
| --- | --- | --- | --- | --- | --- | --- | --- | --- | --- |
| *Paper-intervention Number* | *Intervention type* | **Net income** | | | | **Cost** | | | |
|  |  | *Net income difference (USD)* | *Net income difference (USD ha^-1^)* | *Net income impact* | *Statistical significance* | *Cost difference (USD)* | *Cost difference (USD ha^-1^)* | *Cost impact* | *Statistical significance* |
| 11/1 | Organic nutrient source | 455 | NA | Positive | NA | 25 | NA | Negative | NA |
| 11/2 | Organic nutrient source | 2,131 | NA | Positive | NA | 150 | NA | Negative | NA |
| 16/1 | Crop diversity | NA | 4,591.63 | Positive | NA | NA | NA | NA | NA |
| 22/1 | Legumes | 326 | NA | Positive | NA | 183 | NA | Negative | NA |
| 22/2 | Legumes | 58 | NA | Positive | NA | 114 | NA | Negative | NA |
| 32/1 | Agroforestry | NA | 592.3 | Positive | Yes | NA | NA | NA | NA |
| 36/1 | Legumes | NA | 179 | Positive | NA | NA | 287 | Negative | NA |
| 36/2 | Crop diversity | NA | -115 | Negative | NA | NA | 125 | Negative | NA |
| 41/1 | Livestock integration | 0.44 | NA | Positive | NA | NA | NA | NA | NA |
| 51/1 | Organic farming | NA | 1,314.66 | Positive | Yes | NA | 2,295.44 | Negative | Yes |
| 52/1 | Organic farming | NA | NA | NA | NA | NA | NA | Mixed | Yes |
| 52/2 | Organic farming | NA | NA | NA | NA | NA | NA | Mixed | Yes |
| 67/1 | Organic farming | NA | NA | NA | NA | -165.04 | NA | Positive | NA |
| 104/1 | Bioprotection | NA | -514.74 | Negative | NA | NA | 248.93 | Negative | NA |
| 110/1 | Push-pull/companion crops | 123.72 | NA | Positive | Yes | 3.38 (per capita expenditure) | NA | Neutral | No |
| 129/1 | Landscape structure | -230 | NA | Negative | NA | -19 | NA | Positive | NA |
| 129/2 | Organic farming | -4,461 | NA | Negative | NA | 3,961 | NA | Negative | NA |
| 130/1 | IPM | NA | 1,100.29 | Positive | NA | NA | NA | NA | NA |
| 130/2 | IPM | NA | 1,543.8 | Positive | NA | NA | NA | NA | NA |
| 134/1 | IPM | NA | 757.5 | Positive | Yes | NA | 129 | Mixed | Yes |
|  | **Average:** | **-199.61** | **1,049.94** |  |  | **606.99** | **617.07** |  |  |

| **Table S8:** Crop yield summary statistics for single crops or crops grown in a system (e.g., rotation, intercropping). Values in parentheses represent frequency counts of reported responses. | | | | | | | | | | | |
| --- | --- | --- | --- | --- | --- | --- | --- | --- | --- | --- | --- |
| *Crop* | **Single product** | | | | | | **System product** | | | | |
|  | *Average yield difference (tons ha^-1^ yr^-1^)* | *Average site-years* | *Response count (total)* | *Statistically significant responses (total)* | *Impact* | *Average yield difference (tons ha^-1^ yr^-1^)* | | *Average site-years* | *Response count (total)* | *Statistically significant responses (total)* | *Impact* |
| Potato | 0.2 | 1 | 1 | 0 | Neutral (1) | -6.5 | | 4.3 | 3 | 1 | Neutral (2), Negative (1) |
| Maize | 1.054 | 7.33 | 25 | 16 | Positive (16), Neutral (7), Negative (2) | 0.36 | | 3.93 | 14 | 3 | Positive (8), Neutral (6) |
| Cabbage | 0.11 | 8 | 1 | 1 | Positive (1) | -11.6 | | 6 | 1 | 0 | Negative (1) |
| Cotton | 0.121 | 9 | 1 | 1 | Positive (1) | -0.186 | | 2 | 1 | 1 | Negative (1) |
| Kale | NA | NA | NA | NA | NA | -1.9 | | 6 | 1 | 1 | Negative (1) |
| Swiss chard | NA | NA | NA | NA | NA | -0.4 | | 6 | 1 | 0 | Negative (1) |
| Cowpea | 1.78 | 78 | 1 | 1 | Positive (1) | NA | | NA | NA | NA | NA |
| Millet | 0.8 | 1 | 2 | 0 | Positive (2) | 0.08 | | 4 | 2 | 2 | Positive (1), Negative (1) |
| Soybean | 0.213 | 22 | 3 | 2 | Positive (2), Neutral (1) | -0.135 | | 2 | 1 | 0 | Neutral (1) |
| Rice | 0.569 | 1.5 | 2 | 2 | Positive (1), Negative (1) | 2.1 | | 3 | 1 | 1 | Positive (1) |
| Beans | 1.39 | 4 | 2 | 2 | Positive (2) | -0.136 | | 5 | 2 | 0 | Neutral (2) |
| Spinach | NA | NA | NA | NA | NA | 32.12 | | 2 | 1 | 1 | Positive (1) |
| Wheat | NA | NA | NA | NA | NA | -0.6 | | 2 | 1 | 1 | Negative (1) |
| Groundnut | NA | NA | NA | NA | NA | 1.54 | | 2 | 2 | 1 | Positive (2) |
| Oilseed crop | 0.39 | 4 | 1 | 1 | Positive (1) | -0.0105 | | 4 | 1 | 1 | Negative (1) |
| Other cereals | 0.03 | 49 | 1 | 0 | Neutral (1) | NA | | NA | NA | NA | NA |
| Other fruits (tree) | 3.8 | 1 | 1 | 1 | Positive (1) | 0.3 | | 14 | 1 | 1 | Positive (1) |
| Other fruits (non-tree) | 7.33 | 10.4 | 5 | 5 | Positive (5) | NA | | NA | NA | NA | NA |
| Other vegetables (above-ground) | 15.375 | 5 | 4 | 3 | Positive (3), Neutral (1) | NA | | NA | NA | NA | NA |
| Milk | 1,027 L/head/yr | 1 | 1 | 1 | Positive (1) | NA | | NA | NA | NA | NA |
| Milk | 4.45 t | 9 | 1 | 0 | Neutral (1) | NA | | NA | NA | NA | NA |

| **Table S9:** Greenhouse gas summary statistics by individual interventions and intervention type. For statistical significance, yes = statistically significant, no = not statistically significant, as reported in the respective study. NA = not applicable. | | | | | |
| --- | --- | --- | --- | --- | --- |
| Paper-intervention No. | Intervention type | Change in GHG emissions | Unit | GHG impact | Statistical significance |
| ***CO_2_e*** | | | | | |
| 27/1 | Livestock integration | 6.3 | t CO_2_e year^-1^ | Negative | Yes |
| 56/1 | Other | NA | kg CO_2_e ha^-1^ season^-1^ | Positive | NA |
| 56/2 | Other | NA | kg CO_2_e ha^-1^ season^-1^ | Positive | NA |
| ***CO_2_*** | | | | | |
| 72/1 | Organic nutrient source | NA | NA | Positive | Yes |
| ***N_2_O*** | | | | | |
| 10/1 | Organic nutrient source | 0.03 | kg N_2_O-N ha^-1^ | Neutral | No |
| 10/2 | Organic nutrient source | 0.27 | kg N_2_O -N ha^-1^ | Negative | Yes |
|  | **N_2_O Average:** | **0.15** | kg N_2_O -N ha^-1^ |  |  |

**Figure S1:** Count of significant positive (green), significant negative (red), neutral (i.e., not significant finding; gray) and mixed (yellow) responses to separate agroecological interventions indicators (a, c) and overall categories (b, d) with (a, b) or without (c, d) the presence of local adaptation (i.e., local or indigenous knowledge, extension and education, altering technology by context, and involvement of farmer organization).
